# Preoperative Chemoradiotherapy in Elderly Patients with Locally Advanced Rectal Cancer

**DOI:** 10.1155/2013/610786

**Published:** 2013-12-12

**Authors:** Francesca De Felice, Daniela Musio, Luciano Izzo, Federico Pugliese, Paolo Izzo, Antonio Bolognese, Vincenzo Tombolini

**Affiliations:** ^1^Department of Radiotherapy, Policlinico Umberto I “Sapienza” University of Rome, Viale Regina Elena 326, 00161 Rome, Italy; ^2^Department of Surgery “Pietro Valdoni”, Policlinico Umberto I “Sapienza” University of Rome, Viale Regina Elena 326, 00161 Rome, Italy; ^3^Spencer-Lorillard Foundation, Viale Regina Elena 262, 00161 Rome, Italy

## Abstract

*Purpose*. To evaluate the treatment tolerance and clinical outcomes in patients aged 70 and older with locally advanced rectal carcinoma treated with multimodality approach. *Methods and Materials*. We retrospectively analysed 20 consecutive elderly patients, with histologically proven rectal adenocarcinoma, staged T3-4, and/or node-positive tumour, who received chemoradiotherapy and proceeded to surgical approach. Performance status score and adult comorbidity evaluation-27 score were calculated, and their influence on treatment tolerance and clinical outcomes was analysed. *Results*. All patients completed programmed chemoradiotherapy treatment. Gastrointestinal toxicity was the most common acute side effects: proctitis in 70% of patients and diarrhoea in 55%, classified as Grade 3 in 3 patients only. Radiation dermatitis was reported in 7 patients (35%) and it was graded G3 in one patient. There was no haematological toxicity. Eighteen patients out of 20 underwent surgery. Sphincter preservation was assured in 13 patients. Comorbidity index was related to higher severe acute toxicity (*P* = 0.015) but no influenced treatment outcomes. *Conclusion*. Treatment tolerance with combined modality is good in elderly patients. Due to age, no dose reduction for radiation therapy and chemotherapy should be considered.

## 1. Introduction

Survival and disease control for rectal cancer depend on both degree of wall invasion of the primary tumour and nodal involvement [1]. Preoperative chemoradiotherapy is nowadays used as part of the therapeutic approach for patients with locally advanced rectal cancer to reduce the risk of local recurrence, improve the R0 resection, and preserve the sphincter function [2]. To improve these aims, in the last 10 years, four important randomized published studies—CAO/ARO/AIO-04 [3], STAR-01 [4], ACCORD 12/0405-Prodige 2 [5], and NSABP R-04 [6]—have investigated the optimal neoadjuvant combined-modality treatment with fluoropyrimidine alone (5-FU) or in combination with oxaliplatin (OXP) [7]. Waiting for longer follow-up data from these trials, although concomitant radiation therapy plus 5-FU-based chemotherapy remains the standard of care in rectal cancer, the concept of combination of 5-FU and OXP still seems promising, as described in our previous study [8]. But considering the restrictive selection criteria of randomized studies [3–6], the risks of concomitant treatment in elderly patients have not been adequately characterised in these trials: patients aged over 70 comprise a small part of the entire cohort therefore, the safety and efficacy data of preoperative chemoradiotherapy in elderly population are lacking [9].

The purpose of the present study was to analyze the treatment tolerance and the outcome of preoperative chemoradiotherapy in elderly patients with locally advanced rectal cancer. It is hoped that these data will be potentially useful as a reference in the future for a solid scientific evidence.

## 2. Materials and Methods

### 2.1. Patients Population

This retrospective analysis recruited elderly patients (aged ≥70 years) with locally advanced rectal cancer treated with neoadjuvant intensified chemoradiotherapy. It is a subgroup analysis of our previous study [8] based on the addition of OXP in the neoadjuvant setting to investigate its survival benefit. The study was approved by the Institutional Reviewed Board and patients signed an informed consent. Selection criteria for analysis cases included the following patients: the elderly (≥70 years); those with newly diagnosed histologically proven rectal adenocarcinoma without metastasis; those with stage IIa-IIIc disease (according to the 7th American Joint Committee on Cancer Staging System [10]), where patients staged with a prior edition staging system were restaged according to the 7th edition; those without history of previous radiation therapy or chemotherapy. We used the adult comorbidity evaluation-27 (ACE-27) score, a 27-item validated comorbidity index, for the analysis of patients comorbidities [11]. Patients' performance status was assessed by ECOG Performance status (PS) score [12].

### 2.2. Treatment Protocol

All patients were treated with a long course RT-CHT. Radiation therapy was delivered with a 3D-conformational multiple field technique at a dose of 45 Gy (in 25 daily fractions of 1,8 Gy given in 5 weeks) to the whole pelvis plus a 5,4–9 Gy (in 3–5 daily fractions of 1,8 Gy) to the tumour volume, with 6–15 MV energy photons. Chemotherapy consisted of 2-hour OXP infusion 50 mg/m^2^ on the first day of each week of radiotherapy and five daily continuous infusions of 5-FU 200 mg/m^2^/die. Surgery was scheduled 7–9 weeks after the end of RT-CHT treatment. The type of surgery was left to the surgeon's discretion, and the type of adjuvant chemotherapy was chosen by the oncologist.

### 2.3. Patient Evaluation and Followup

During treatment patients were evaluated daily. Toxicity was evaluated using National Cancer Institute's Common Terminology Criteria for Adverse Events version 3.0 [13]. Pathological staging (ypTN), radial margins, downsizing, and downstaging were recorded. Overall survival (OS) and disease-free survival (DFS) were measured in months from the end of the neoadjuvant treatment. After surgery, all patients were monitored at three-month intervals for the first year and at six-month intervals for the subsequent years. Follow-up data were updated in March 2013.

### 2.4. Statistical Analysis

The following end-points were examined: toxicity, compliance to treatment, OS and DFS. To determine the influence on treatment tolerability the variable ACE-27 was considered and the univariate analysis was performed using the non-parametric Bernard test. Statistical tests were one-sided. Statistical analysis was performed using MATLAB software, version 7.5.0.342 (R2007b).

## 3. Results

### 3.1. Patient Characteristics

Between February 2007 and December 2011, 20 elderly patients were enrolled: five females and 15 males; the average age was 73.1 years (range 70–76 years); the average ACE-27 score was 0.7 (range 0–2) and PS score 0.4 (range 0–2). Demographic characteristics of patients enrolled are listed in Table 1.

### 3.2. Treatment Compliance

All elderly patients completed programmed RT-CHT treatment. No patients had decreased chemotherapy dose. Only 4 patients interrupted radiation therapy for an average period of 15.5 days (range 6–22) due to acute toxicity.

### 3.3. Toxicity

Incidences of major acute and late toxicities are listed in Table 2. Gastrointestinal toxicity was the most common acute side effects: proctitis in 70% of patients and diarrhoea in 55%, classified as Grade 3 in 3 patients only. Radiation dermatitis was reported in 7 patients (35%) and it was graded G3 in one patient. No severe neurological and renal toxicity were seen. Late toxicity was assessed and involved the following conditions: faecal incontinence, dermatitis, proctitis, and venous thrombotic events (VTEs). In total, the incidence rate of any late toxicity was 30% (6/20). Excluding the single case of VTE, severe late toxicity was not recorded.

In univariate analysis, ACE-27 >0 had no influence on treatment tolerability (*P* value = 0.28), whereas we found that the rate of severe acute toxicity was higher in patients with ACE-27 scores > 1 (100% versus 22.2%; *P* value = 0.015).

### 3.4. Treatment Response

18 patients out of 20 underwent surgery. We recommended a “wait and see” approach to one patient with imaging documented clinical complete response after neoadjuvant treatment due to ACE-27 = 1. One patient had a myocardial infarction two weeks after the end of chemoradiotherapy and he died. Sphincter preservation was guaranteed in 77.78% of patients. Low anterior resection was performed in 11 patients, TEM in 2 patients, and Miles' surgery in 4 patients. One patient died of intraoperative complications. An involvement of radial margin was never present. 17 patients (94.4%) had some form of downstaging from preoperative treatment. Pathological complete response (pCR), defined as the absence of any residual tumoural cells detected in the operative specimen, was recorded in 3 patients (16.6%).

### 3.5. Survival

Median follow-up period was 44 months (range 16–71 months). In total, OS was 85% and DFS was 66.7%. Only one death was detected during the follow-up period, and it was cancer-related. Four patients were followed for at least 60 months and they are still disease-free survivals. In total, six patients had progressed disease after treatment. Distant metastases were higher than local recurrences (66.6% versus 33.3%) (Table 3). ACE-27 >0 had no influence on OS and DFS (*P*-value = 0.08 and 0.21, resp.).

## 4. Discussion

Colorectal cancer (CRC) is a common malignancy, with approximately half the cases occurring in patients older than 70 years [14]. The choice of the optimal management for elderly patients with CRC is a complicated procedure, especially in locally advanced rectal cancer, due to the multimodalities treatment approaches available [15]. Preoperative chemoradiotherapy (RT-CHT), based on 5-FU, and total mesorectal excision (TME) surgery are considered the standard treatment for patients with clinical stage ≥IIA rectal cancer. It has been extensively demonstrated that this multimodality treatment improves local control, toxicity (acute and chronic), and sphincter preservation [16–23]. The value of the polichemiotherapic regime (OXP plus 5-FU) in association with the standard long course radiation therapy, is still controversial. The results of the randomized studies were discordant. A pCR improvement was observed with the introduction of OXP, in the CAO/ARO/AIO-04 trial [3]; whereas the studies STAR-01 [4], ACCORD 12/0405-Prodige 2 [5], and NSABP R-04 [6] had not shown significant benefit. However, due to an increase in life expectancy, elderly represents a large group of rectal oncological patients. But they are usually underrepresented or excluded from clinical trials because of the restrictive age inclusion criteria of the studies design. As a consequence of the little recruitment of elderly patients in clinical trial, our judgment of the appropriateness of therapeutic strategy is severely inadequate. Considering that in the literature there is no clear definition of “elderly” patients and that the chronological age could be very different from biological age [9], the choice of initial treatment should be influenced mostly by comorbidities and potential therapy side effects [24]. The last published ESMO Consensus Guidelines have been explicitly detailed that there is no age limit for the choice of treatment strategy in elderly patients with rectal cancer [2]. Nevertheless, the real role of neoadjuvant RT-CHT in this population is still unclear. Several studies have evaluated RT-CHT in elderly patients, but the results of these studies were discordant [25–28]. Our analysis aimed to clarify the tolerability and efficacy of concomitant treatment in elderly locoregionally advanced rectal cancer patients. Our data showed that patients could tolerate the full cycle of chemotherapy and the radiation total dose prescribed without any modifications of the planning treatment. In previous studies, dose reduction of radiation therapy or chemotherapy was required in 4.2–37.3% [25–27]. The rates of severe acute and late toxicities in our analysis were better than those of previously published clinical trials [24–27], and according to those studies, we found that gastrointestinal toxicity and skin reactions were more represented. Moreover, the rates of pCR (16.6%) and sphincter preservation (77.78%) were higher than other trials (0.8–16.6% and 52–64%, resp.). And, comparing survival rates, same better evidences were manifested. The 2-year OS was 84% in Tougeron et al. trial [25]; the 3-years OS rates was 49.9% in Margalit et al. study [27], and Cai et al. [28] reported a 3-year OS of 81.5% in patients with curative intent treatment. In our study, we found that RT-CHT, based on OXP and 5-FU, could still improve 5-year OS and DFS: patients followed for at least 60 months are still disease-free survivals. According to Margalit et al. [27], ACE-27 comorbidity index was related to higher severe acute toxicity but no influenced treatment outcomes. Moreover, there was no association between increasing ACE-27 score and treatment modification.

The major limitation of this study was that it was a retrospective analysis with a small sample. But all patients were submitted to the same treatment schedules. However, this analysis focused on a significant topic concerning the fleasibility and the efficacy of neoadjuvant RT-CHT for elderly patients with locally advanced rectal cancer. A prospective randomized study with a large sample should be conducted to confirm preliminary results.

## 5. Conclusions

Elderly patients should be exposed to the same treatment received by younger patients. Our analysis had shown that neoadjuvant RT-CHT, based on oxaliplatin and 5-FU, for patients aged ≥70 is safe and well tolerated. High number of cases could confirm these results, but it should be considered an effective treatment to improve local control and overall survival in elderly rectal cancer patients.

## Figures and Tables

**Table 1:** Patients' characteristics.

Characteristic	Patients (%)
Age	
Average (range)	73.1 (70–76)
Gender	
Female	5 (25)
Male	15 (75)
ACE-27 score	
0	8 (40)
1	10 (50)
2	2 (10)
PS score	
0	13 (65)
1	6 (30)
2	1 (5)
Histology	
Adenocarcinoma	20 (100)
Clinical stage	
IIA	2 (10)
IIIB	11 (55)
IIIC	7 (35)
Neoadjuvant treatment	
RT-CHT intensified	20 (100)

RT: radiation therapy; CHT: chemotherapy.

**Table 2:** Patients acute and late toxicities.

Acute toxicity	G1	%	G2	%	G3	%
Allergy immunology						
Allergic reaction hypersensitivity	1	5				
Constitutional symptoms						
Fatigue	3	15	1	5		
Fever	1	5				
Dermatology skin						
Rash desquamation			4	20		
Radiation dermatitis	2	10	1	5	1	5
Gastrointestinal						
Constipation			4	20		
Diarrhoea	7	35	2	10	1	5
Nausea	2	10				
Proctitis	2	10	10	50	3	15
Neurology						
Neuropathy: sensory	4	20	1	5		
Pain						
Abdominal pain or cramping	2	10			1	5
Renal genitourinary						
Dysuria-painful urination	2	10	3	15		

	G3	%	G4	%	G5	%

Cardiovascular						
VTE	1	5				

Late toxicity	G1	%	G2	%	G3	%

Gastrointestinal						
Proctitis	4	22.2				
Faecal incontinence	1	5.5				
Dermatology skin						
Radiation dermatitis	1	5.5				

**Table 3:** Survival details.

Detail	Percentage
Survival	
Overall	85%
Disease free	66.7%
Cancer-related death	5.5%
Recurrence	
Local	33.3%
Distant	66.6%
